# Changes in erythrocyte polyunsaturated fatty acids and plasma eicosanoids level in patients with asthma

**DOI:** 10.1186/s12944-018-0853-y

**Published:** 2018-09-01

**Authors:** Jing Zhou, Lifang Chen, Zhenjie Liu, Ling Sang, Yimin Li, Dongjuan Yuan

**Affiliations:** 1 Department of Respiratory and Critical Care Medicine, the First Affiliated Hospital of Guangzhou Medical University, Guangzhou Institute of Respiratory Health, Guangzhou, 510120 China; 2 Department of Respiratory, The affiliated hospital of Guangdong medical University, Zhanjiang, 524023 China; 3 The Second Affiliated Hospital of Guangdong University of Chinese Medicine, Guangzhou, 510120 China; 4 College of Veterinary Medicine, South China Agricultural University, No.483 Wushan Rd, Guangzhou, 510642 People’s Republic of China; 5 Department of Biochemistry, Guangdong Medical University, Zhanjiang, 524023 China

**Keywords:** Asthma, Polyunsaturated fatty acids, Eicosanoids, Inflammation

## Abstract

**Background:**

To investigate the changes of polyunsaturated fatty acids (PUFAs) and their downstream eicosanoids in patients with asthma, the levels of erythrocyte membrane lipids and plasma lipid metabolites were examined.

**Methods:**

Erythrocyte membrane lipids were extracted and esterificated, and then fatty acid compositions were determined by gas chromatography. The concentrations of six eicosanoids of PGE_2_, TXA_2_, LTB_4_, PGE_1_, 6-k-PGF_1**α**_ and PGF_2**α**_ in plasma were measured by ELISA.

**Results:**

The results showed that the contents of erythrocyte membrane fatty acids in patients with asthma were mainly composed of C16:0, C18:0, C18:1, C18:2(n-6), and C20:4(n-6). The ratio n-6/n-3 PUFAs in patients and health persons were (4.42 ± 1.33):1 and (3.21 ± 0.79):1 (*p* < 0.01), showing statistically significant differences. ELISA results showed that the levels of plasma PGE_2_, TXB_2_, and PGE_1_ in patients were higher than health persons; and the levels of eicosanoids of PGF_2α_ and 6-k-PGF_1α_ were significantly lower in patient group than healthy group (*p* < 0.05), but LTB_4_ was no obvious difference (*p* = 0.09). Increased ratio of n-6/n-3 PUFAs is consistent to the increased levels of pro-inflammatory PGE_2_ and TXB_2_ and anti-inflammatory PGE_1_ originated from C20:4(n-6) and C18:2(n-6), indicating that increased ratio of n-6/n-3 PUFAs and eicosanoids from n-6 PUFAs might promote the progress of airway inflammation of asthma.

**Conclusion:**

Changes of erythrocyte fatty acids, n-6/n-3 PUFAs ratio and the levels of plasma PGE_2_, TXB_2_, and PGE_1_ in patients with asthma were relevant to airway inflammation in some extent. Therefore, it could be proposed that increase of n-3/n-6 PUFAs ratio by diet supplementation of n-3 PUFAs might effectively improve airway inflammation in asthma.

## Background

Asthma is an allergic disease of the airways associated with airway hyperresponsiveness to various bronchoconstrictor stimuli [1, 2]. The incidence of asthma has rapidly increased in China with the economic and industrial development in the last decades; this disease becomes one of the most common chronic diseases in China [3]. This epidemic trend has been regarded as correlation with lifestyle changes, such as hygiene, an indoor life, reduced physical activity, and a modified diet. There are some diet hypothesis of an unbalance high intake of n-6 polyunsaturated fatty acids (n-6 PUFAs) such as linoleic acid (LA, C18:2) and arachidonic acid (AA, C20:4), may contribute to the incidence of allergic sensitization and asthma [4].

PUFAs are important components of cellular membrane and functional lipid mediators, which can be divided into n-6 PUFAs and n-3 PUFAs [4]. PUFAs can be metabolized into pro-inflammatory or anti-inflammatory mediators of prostaglandins (PGs), thromboxanes (TXs), leukotrienes (LTs), and lipoxins. These eicosanoids form a complex network to play a regulatory role in cell chemotaxis, inflammation, vascular permeability [4]. Airway inflammation is a common pathological feature of all types of asthma, which is characterized by airway epithelial injury, and results in morpho-functional modifications [2].

**Fig. 1:**
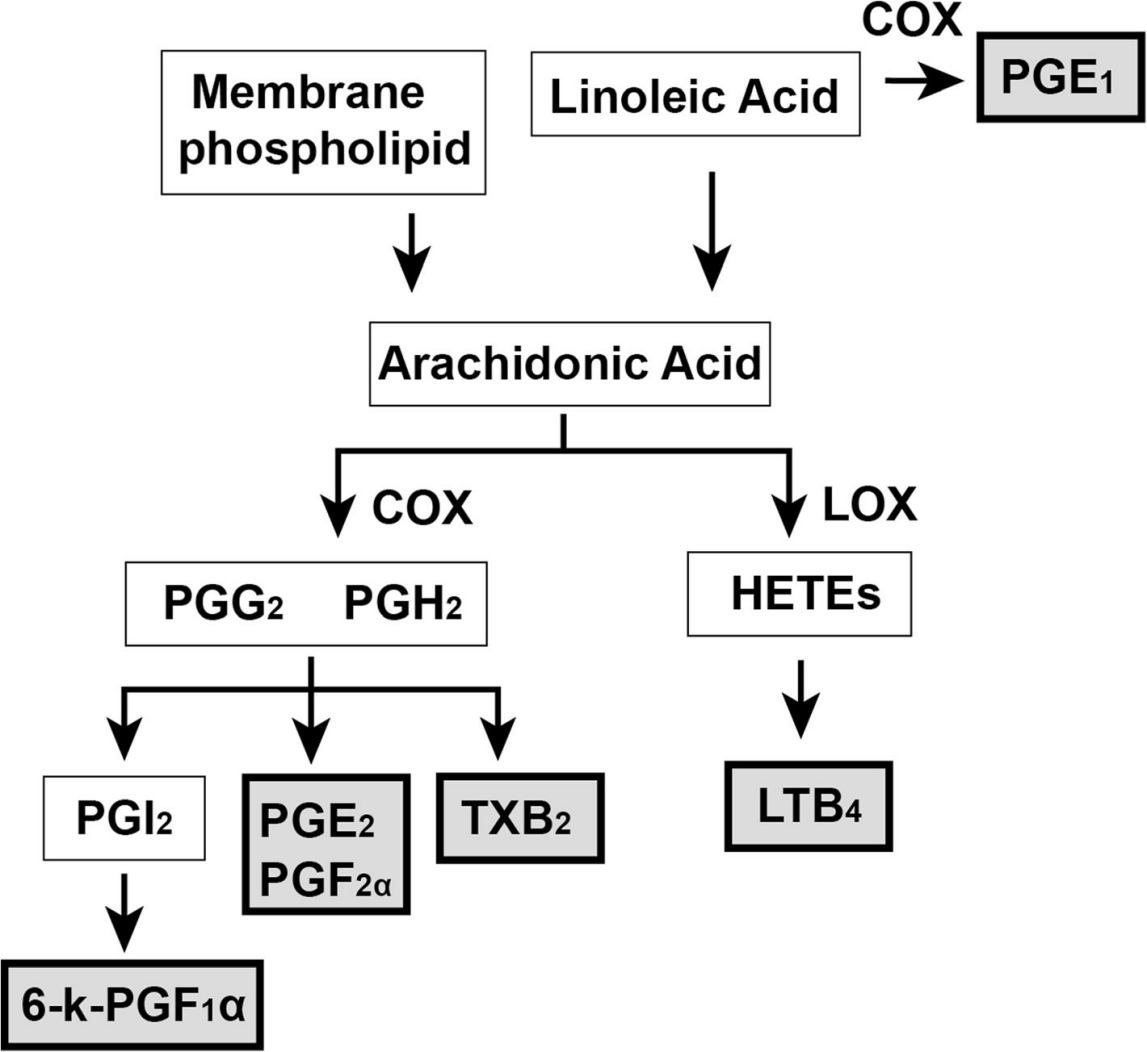
Biosynthesis of detected eicosanoids from PUFAs

In this study, we detected the composition of fatty acids in erythrocyte membrane and downstream eicosanoids in plasma including prostaglandin E2 (PGE_2_), prostaglandin E1 (PGE_1_), thromboxane B2 (TXB_2_), leukotriene B4 (LTB_4_), prostaglandin F1a (PGF_1a_), prostaglandin F2a (PGF_2a_) (Fig. 1) in patients with asthma and healthy persons**,** so as to explore the changes of these lipids in asthma and elucidate their correlations.

## Methods

### Subjects, study design

Thirty-one subjects with physician-diagnosed asthma were recruited from the first affiliated hospital of Guangdong medical university. A group of healthy subjects were recruited from Blood center of Zhanjiang in Guangdong province. All subjects were recruited between March 2008 and September 2008. This study goal was to test the compositions of erythrocyte fatty acids and plasma eicosanoids in patients with asthma and healthy persons.

### Blood samples collection

The blood samples were collected to separate the erythrocytes and stored at − 80 °C until analyses. Ten percent blinded duplicates were included for quality control purposes for fatty acids and eicosanoids’ assays in blood.

### Fatty acid methylation

0.5 ml of heparinized venous blood samples were centrifuged for 20 min in 3000 rpm/min at 4 °C, and then the supernatants were removed in order to remove leukocytes and platelets. The lower layer cells were washed by double deionized water, and then centrifuged for 10 min in 3000 rpm/min to remove the supernatants. This was repeated three times to collect the lower layer of erythrocytes. In order to analyze the compositions of erythrocyte fatty acids, a simplified method for analysis of PUFAs was used to extract total lipids and fatty acid methylation [5]. One milliliter (ml) n-hexane and 1 ml 14% boron trifluoride (BF_3_)/methanol (MeOH) (Sigma-Aldrich) were added to silica tube. Tricosanic acid (C17:0) (0.02 mg: 100 μl, 0.2 mg/ml) was also added to each tube as internal standard. Air was purged with a gentle stream of nitrogen. Samples were methylated for 1 h at 100 °C and the reaction stopped with the addition of 1 ml double deionized water. After centrifugation for 10 min at 3000 g, the upper hexane layer containing the fatty acid methyl esters was transferred to a 2 ml GC vial [6].

### GC analysis

**Fig. 2:**
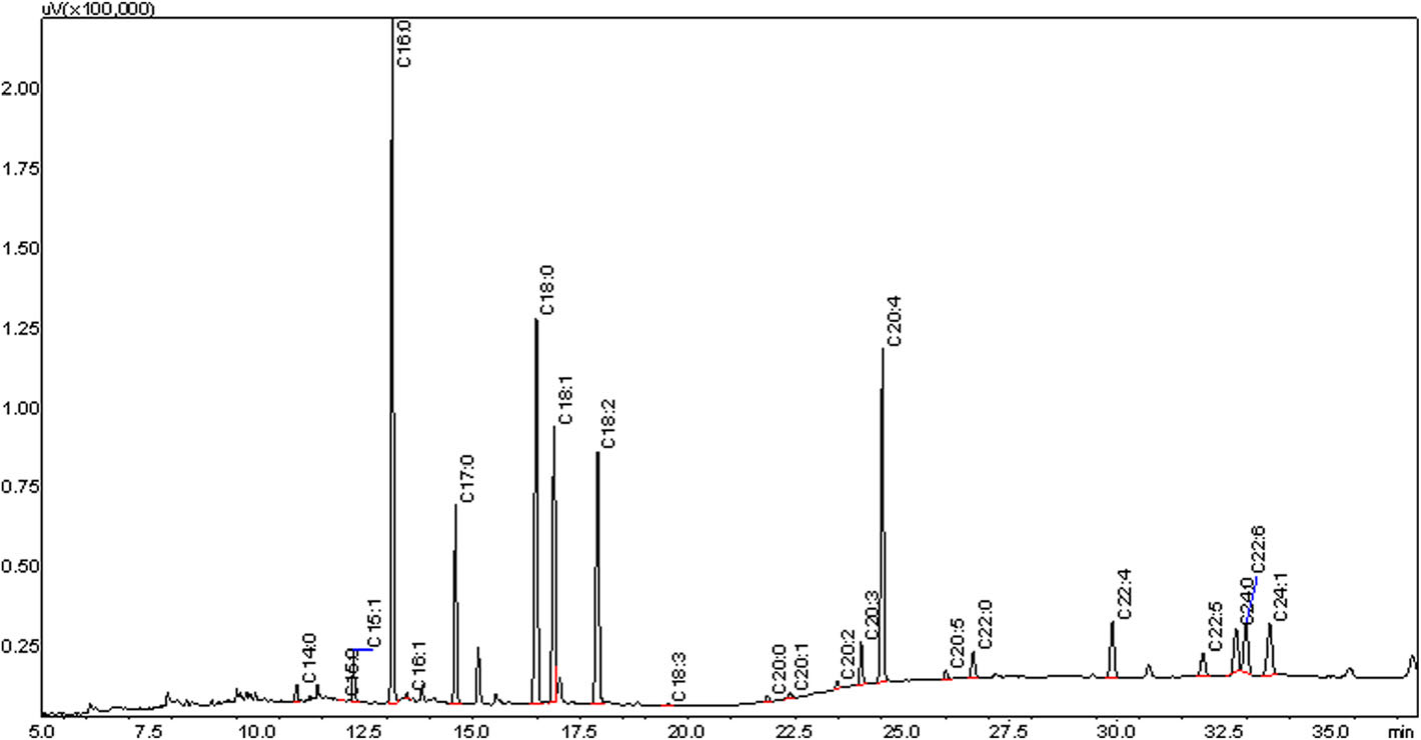
Gas chromatogram of blood fatty acids in asthma patient

Fatty acid methyl esters were separated on a Shimadzu Scientific Instruments, GC-2010 gas chromatograph equipped with a flame ionization detector and 30 m × 0.25 mm × 0.25 m Omega-WAX capillary column (J&W, Agilent). Helium was used as the carrier at a constant flow rate of 10.1 ml/min. One microliter fatty acid methyl esters were injected into the column in split mode. Fatty acid methyl esters were eluted using a temperature program from 100 to 250 °C. The initial temperature of 100 °C ramped to 220 °C at 15 °C/min and held for 12 min, then ramped to 250 °C at 10 °C/min and held for 13 min. Fatty acid peaks were identified by comparing the retention time of each peak against the retention times of validated fatty acid standards (Supelco, Bellefonte, PA) (Fig. 2). Unidentified peaks were not included in the calculation of the percentage in total fatty acid [5, 6]. Values are expressed as % total fatty acids, and total fatty acid concentration (nmol/g viscera) was calculated by comparison of gas chromatography peak areas relative to that of the C17:0 internal standard.

### Quantification of lipid mediators

Six kinds of eicosanoids derived from C18:2 and C20:4 were detected. Immunoassays for PGE_2_, PGE_1_, TXA_2_, LTB_4_, PGF_1a_, and PGF_2a_ were performed in duplicate using the human PGE_2_ Quantikine ELISA kit, human PGE_1_ Quantikine ELISA kit, human TXA_2_ Quantikine ELISA kit, human LTB_4_ Quantikine ELISA kit, human PGF_1a_ Quantikine ELISA kit, and human PGF_2a_ Quantikine ELISA kit (R&D Systems), respectively. Plasma samples were assayed according to the manufacturer’s instructions. Briefly, plasma samples and controls were added to ELISA plates pre-coated with the capture antibody, then incubated in 37 °C for 60 min, washed and detected based on a colorimetric reaction between a horseradish peroxidase-labeled detection antibody and the tetramethyl benzidine substrate solution. The color absorbance at 450 nm was measured using a spectrophotometer (Bio-Rad).

### Statistical analysis

T-test and one-way ANOVA analyses were used to evaluate differences in mean erythrocyte fatty acids and mean eicosanoids’ concentrations. Spearman’s correlation coefficient and partial correlation coefficient were used to describe the concentrations of erythrocyte fatty acid and plasma eicosanoids (Tables 1 and 2). Analyses were performed with SPSS 16.0, a *p* < 0.05 was considered statistically significant.

**Table 1:** Comparison of fatty acid compositions in healthy persons and patients (%, X ± S)

	Healthy group (*n* = 31)	Patients group (*n* = 31)
C14:0	0.30 ± 0.25	0.65 ± 0.48**
C15:0	0.10 ± 0.04	0.17 ± 0.09
C15:1	2.43 ± 0.46	1.95 ± 0.44**
C16:0	17.76 ± 0.83	19.74 ± 2.12**
C16:1	0.26 ± 0.12	0.51 ± 0.17**
C18:0	10.26 ± 0.60	10.43 ± 0.69
C18:1	12.14 ± 1.23	14.02 ± 1.96**
C18:2(n-6)	10.69 ± 1.24	10.47 ± 1.99
C18:3(n-3)	0.07 ± 0.02	0.09 ± 0.07
C20:0	0.32 ± 0.05	0.34 ± 0.10
C20:1	0.25 ± 0.05	0.27 ± 0.08
C20:2(n-6)	0.79 ± 0.46	0.30 ± 0.13**
C20:3(n-6)	1.37 ± 0.30	1.45 ± 0.32
C20:4(n-6)	16.52 ± 1.07	15.52 ± 2.12*
C20:5(n-3)	0.64 ± 0.35	0.73 ± 0.41
C22:0	4.86 ± 1.93	4.16 ± 2.74
C22:4(n-6)	2.96 ± 0.54	2.76 ± 0.80
C22:5(n-3)	2.25 ± 0.40	2.18 ± 0.48
C22:6(n-3)	7.57 ± 1.52	4.55 ± 1.87**
C24:0	4.24 ± 1.03	6.21 ± 1.55*
C24:1	4.21 ± 0.70	3.51 ± 1.12**
n-6	32.33 ± 1.57	30.49 ± 3.03**
n-3	10.52 ± 2.05	7.56 ± 2.37**
n-6/n-3	3.21 ± 0.79	4.42 ± 1.33*
AA	16.52	15.52
EPA + DHA	8.21	5.28
AA/(EPA + DHA)	2.01	2.94
SFA	37.84	41.70
MUFA	19.29	20.26
PUFA	42.86	38.05
UFA	62.15	58.31
SFA/UFA	0.61	0.72

Note: *, *p* < 0.05; **, *p* < 0.01

**Table 2:** Comparison of the concentrations of 6 eicosanoids in healthy and patient groups (ng/ml, pg/ml for LTB_4_, X ± S)

	PGE_2_ (*n* = 31)	TXB_2_ (*n* = 30)	LTB_4_ (*n* = 30) (pg/ml)	PGE_1_ (*n* = 31)	6-k-PGF_1α_ (*n* = 28)	PGF_2α_ (*n* = 22)
Healthy group	5.09 ± 1.47	4.28 ± 0.58	2.78 ± 0.56	2.96 ± 0.46	1.40 ± 0.63	1.84 ± 0.84
Patients group	5.99 ± 0.87**	4.88 ± 0.89**	3.02 ± 0.64	4.54 ± 0.48**	0.94 ± 0.36**	1.11 ± 0.73*

Note: *, *p* < 0.05; **, *p* < 0.01

## Results

### Baseline

Overall, 31 patients and 31 healthy persons were included in this study, with a mean age of 58 ± 6.5 years old (patients group) and 25 ± 2.3 years old (healthy group), respectively. In the patients group, there were 13 men (41.90%), 5 patients in 27–29 years old (16.10%), 9 in 30–39 years old (26.03%), 5 in 40–49 years old (16.10%), 6 in 50–59 years old (19.35%), 5 in 60–69 years old (16.10%), 1 in 70–79 years old (3.22%). Meanwhile, the healthy group was 15 healthy men (48.4%) who aged> 16 years old, with no prior history of asthma or other respiratory diseases, allergic diseases, and other chronic diseases.

### Measured lipids

Comparison of the contents of fatty acids in the erythrocyte membrane between patients group and healthy group was given in Table 1. The compositions of erythrocyte saturated fatty acid (SFAs), PUFAs, and monounsaturated fatty acids (MUFAs) from patients with asthma were 41.70%, 38.05%, and 20.26%, respectively. In the healthy group, the contents of SFAs, PUFAs, and MUFAs were 37.84%, 42.86%, and 20.26%, respectively. Hence, SFAs, mainly composed of C16:0 and C18:0, were the most abundant fatty acids in patients group, while PUFAs, mainly composed of C18:2(n-6) and C20:4(n-6), were the most abundant components in healthy group. The percentages of C14:0, C16:0, and C24:0 in patients with asthma were 0.65 ± 0.48, 19.74 ± 2.12, and 6.21 ± 1.55, which was significantly higher than that of healthy persons of 0.30 ± 0.25, 17.76 ± 0.83, and 4.24 ± 1.03, respectively. Previous study also observed a significant accumulation of saturated fatty acids in chronic bronchitis and chronic obstructive pulmonary disease [7].

The percentages of n-6 and n-3 PUFAs in total erythrocyte fatty acids in patients were 30.49 ± 3.03 and 7.56 ± 2.37, while in healthy group, the percentages of n-6 and n-3 PUFAs were 32.33 ± 1.57 and 10.52 ± 2.05, respectively. The contents of n-6 and n-3 PUFAs were significantly decreased in patients group compared to the healthy group, while the ratio of n-6/n-3 PUFAs in patients group were significantly higher than those in healthy group (*p* < 0.01).

In details, the difference of n-6 PUFAs of two groups was mainly due to the lower percentage of AA in patients group than that of healthy group. As for n-3 PUFAs, the percentages of EPA in patients group was as much as that in healthy group, while DHA in patients group was significantly lower than that in healthy group. Accordingly, the differences of n-3 PUFAs between two groups were mainly caused by the differences in DHA.

**Fig. 3:**
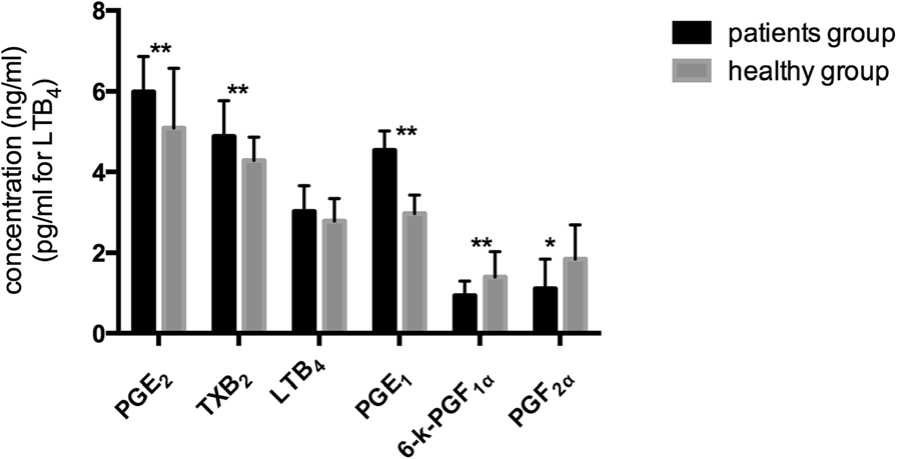
Comparison of the concentrations of 6 eicosanoids in healthy and patient groups (ng/ml, pg/ml for LTB_4_, X ± S)

As shown in Table 2, the concentrations of PGE_2_, TXB_2_, 6-k-PGF_1α_, PGF_2α_ and PGE_1_ in patients group were significantly different to the healthy group. The levels of PGE_2_, TXB_2_, and PGE_1_ in patients group were higher than those in healthy group (*p* < 0.05), while the levels of PGF_2α_ and 6-k-PGF_1α_ in patient group were lower than the healthy group (Fig. 3). The concentrations of LTB_4_ was no significant difference between two groups (*p* > 0.05) (Fig. 3).

## Discussion

Our study showed that erythrocyte fatty acids were mainly composed of C16:0, C18:0, C18:1, C18:2(n-6), and C20:4(n-6) in patients with asthma and healthy persons. The contents of fatty acids from the high to low were SFA, PUFA, and MUFA in patients group, respectively, while the healthy group was PUFA, SFA, and MUFA. The proportion of SFAs in erythrocytes in patients with asthma was higher than that in healthy persons. The contents of C14:0, C16:0, and C24:0 in patients were higher than healthy persons. It is known that accumulation of fatty acids due to altered metabolism could induce cell death, cytokine secretion and activation of inflammatory processes. SFAs have been described as ligands of Toll-like receptor 4 to induce fatty acid-mediated cytotoxicity by activation of nuclear factor kappa B and C-jun N-terminal kinase in murine macrophages and adipocytes [8–11]. Accumulation of SFAs can activate organellar dysfunctions of lysosomal destabilization [12–14] and endoplasmic reticulum stress [15–17] via the intrinsic apoptosis pathway and activation of death receptors of tumor necrosis factor (TNF) receptors and TNF-related apoptosis inducing ligand-receptors via extrinsic apoptotic pathways [18, 19]. C24:0 has also been described to stimulate NADPH oxidase activity to enhance lipid peroxidation in fibroblasts [20]. Thus, the increased level of SFAs, especially C14:0, C16:0, and C24:0, might be associated with the inflammatory responses of asthma.

PUFAs could exert various functions through directly mechanisms or their metabolites. In this study, the proportion of PUFAs in erythrocytes in patients with asthma was lower than that in healthy persons, indicating that increased consumption of PUFAs in asthma might induce the lower level of PUFAs in patients than the healthy persons. As for the significantly difference (58 ± 6.5 vs 25 ± 2.3) in ages from two groups in this study, a number of clinical trials, including patients with asthma, have investigated the changs in the ratio of n-3/n-6 PUFAs ranged from 3 to 72 years old, showing that the age may not influence the results of study [4]. Our data showed that the ratio of n-6 PUFA/n-3 PUFAs (4.42 ± 1.33:1) in erythrocyte in patients with asthma was significantly higher than that in healthy persons (3.21 ± 0.79:1) (*p* < 0.01). n-6 PUFAs could regulate the inflammatory responses of the body by their metabolites [4]. n-3 PUFAs (mainly EPA and DHA) could competitively inhibit the oxidation of n-6 PUFAs by COX or LOX and reduce the production of eicosanoids from n-6 PUFAs [2, 4]. Observational studies and clinical trials have showed that intake of n-3 PUFAs with fish oil supplementation have the protective effect on exercise-induced bronchoconstriction in asthma [21, 22]. Therefore, our data of higher ratio of n-6/n-3 PUFAs is consistent to these clinical trials, indicating that the higher ratio of n-6/n-3 PUFAs could partially reflect the inflammatory status of asthma.

PUFAs could be metabolized to PGs, TXs, and LTs through COX and LOX pathways to involve in regulating inflammatory responses [23]. In our study, the concentration of pro-inflammatory mediators of PGE_2_ and TXB_2_ produced by the action of COX on AA are the predominant pro-inflammatory eicosanoids, were higher in asthma patients than that in healthy persons (Table 2) but 6-K-PGF_1α_ was lower in patients than that in healthy persons. It is consistent to previous study of the higher level of PGE_2_ and TXB_2_ in the plasma of asthma patients than that of healthy persons, and the concentration of 6-K-PGF_1α_ in patients was lower than healthy persons [24]. LTB_4_, the downstream metabolites of AA produced by the action of 5-LOX, has been considered as a pro-inflammatory mediator and induce airway hyper-reactivity [25, 26]. LTB_4_ in BALF fluid or blood from severe asthma were higher than moderate symptomatic asthma patients and normal [27, 28]. However, the concentration of LTB_4_ in two groups showed no statistical difference in this study. PGF_**2α**_ have been also demonstrated to be involved in acute or chronic inflammation in several situations such as asthma [26]. In this study, the concentration of PGF_**2α**_ in patients was lower than the healthy group. Furthermore, the concentration of PGE_1_, a typical anti-inflammatory factor, was also higher in asthma patients than that in healthy persons. PGE_1_ could dilate blood vessels, inhibit oxygen radicals and macrophage activation [3]. Our data showed that the relationship between PUFAs and their downstream metabolites were consistent with inflammation.

Asthma is a complex syndrome with different clinical phenotypes, airway inflammation is a response companied with the pro-inflammatory and anti-inflammatory reactions in the body. In the initial stage of airway inflammation, the contents of pro-inflammatory mediators might be high, while with the progress of inflammatory responses, the contents of anti-inflammatory mediators were also produced to involve in inflammatory responses. Eicosanoids of PGE_2_, TXB_2_, and PGE_1_ might be the potential biomarkers associated with the inflammatory responses of asthma, and the treatment of asthma using n-3 PUFAs may be useful to improve the symptoms of asthma [4].

## Conclusions

In conclusion, we investigated the compositions of erythrocyte fatty acids and plasma eicosanoids in patients with asthma from Zhanjiang. Changes in fatty acids and eicosanoids level, especially the higher level of PGE_2_, TXB_2_, PGE_1_, and the ratio of n-6/n-3 PUFAs in patients with asthma than healthy persons, might be due to the airway inflammation. It provides the basis for studying the association of fatty acids and its metabolites with airway inflammation of asthma.

## Abbreviations

AA: Arachidonic acid
ALA: α linolenic acid
DHA: Docosahexaenoic acid
DPA: Docosapentaenoic acid
EPA: Eicosapentaenoic acid
LA: Linolenic acid
LTB4: Leukotriene B4
MUFA: Monounsaturated fatty acid
PUFA: Polyunsaturated fatty acid
SFA: Saturated fatty acid
